# Altered Fronto-Striatal and Fronto-Cerebellar Circuits in Heroin-Dependent Individuals: A Resting-State fMRI Study

**DOI:** 10.1371/journal.pone.0058098

**Published:** 2013-03-06

**Authors:** Yarong Wang, Jia Zhu, Qiang Li, Wei Li, Ning Wu, Ying Zheng, Haifeng Chang, Jiajie Chen, Wei Wang

**Affiliations:** Department of Radiology, Tangdu Hospital, The Fourth Military Medical University, Xi’an, China; Bellvitge Biomedical Research Institute-IDIBELL, Spain

## Abstract

**Background:**

The formation of compulsive pattern of drug use is related to abnormal regional neural activity and functional reorganization in the heroin addicts’ brain, but the relationship between heroin-use-induced disrupted local neural activity and its functional organization pattern in resting-state is unknown.

**Methodology/Principal Findings:**

With fMRI data acquired during resting state from 17 male heroin dependent individuals (HD) and 15 matched normal controls (NC), we analyzed the changes of amplitude of low frequency fluctuation (ALFF) in brain areas, and its relationship with history of heroin use. Then we investigated the addiction related alteration in functional connectivity of the brain regions with changed ALFF using seed-based correlation analysis. Compared with NC, the ALFF of HD was obviously decreased in the right caudate, right dorsal anterior cingulate cortex (dACC), right superior medial frontal cortex and increased in the bilateral cerebellum, left superior temporal gyrus and left superior occipital gyrus. Of the six regions, only the ALFF value of right caudate had a negative correlation with heroin use. Setting the six regions as “seeds”, we found the functional connectivity between the right caudate and dorsolateral prefrontal cortex (dlPFC) was reduced but that between the right caudate and cerebellum was enhanced. Besides, an abnormal lateral PFC-dACC connection was also observed in HD.

**Conclusions:**

The observations of dysfunction of fronto-striatal and fronto-cerebellar circuit in HD implicate an altered balance between local neuronal assemblies activity and their integrated network organization pattern which may be involved in the process from voluntary to habitual and compulsive drug use.

## Introduction

Drug addiction is viewed as the transitions from a pattern of voluntary drug use to dysfunctional stimulus-response habit or even to compulsive behavioral phenotype [1]. Considerable evidence now attributes the transitions to drug-induced adaptive changes in the brain structure and function. These changes may lead to reorganization of brain microzones and functional networks, presenting with brain structural disturbance, extensive brain functional abnormalities and disrupted networks [2]–[3].

Heroin is a highly addictive drug [4]. Past years witnessed the achievements of neuroimaging studies in revealing the neural substrates underlying heroin addiction process. Specifically, structural MRI discloses the impaired white matter integrity induced by chronic heroin use (CHU) in the right frontal sub-gyral, corpus callosum, thalamic radiation, inferior longitudinal fasciculus [5]–[6], parietal lobule, and insular [7], and the reduced gray matter volume and density within frontal, cingulate and temporal cortices in heroin-dependent individuals [8]–[9]. Task-related fMRI reveals heroin-use-induced abnormal brain responses to external stimuli, including inhibitory control, decision making and stress regulation cognitive functions [10]–[13], and involving in reward, cognitive control, motivation and/or drive and salience evaluation, learning and memory circuitry [14].

Resting-state functional connectivity analysis and networks assessment are more effective in elucidating brain pathophysiologic state both in rest and task state and even in predicting subsequent stimulus-related neural responses from an integrated perspective. For example, through graph theory analysis, Liu et al. identified the dysregulated neural networks which might be contributed to deficits in self-control, inhibitory control and stress regulation [15]. Ma et al. [16] applied seed based correlation analysis verified abnormal functional organization in the neural circuits responsible for increased salience value and reduced cognitive control observed in task-related fMRI in heroin addicted brain. Moreover, they demonstrated a abnormal default mode network functional connectivity which may involved in addiction-related enhanced memory processing and decreased attention and self-monitoring using independent component analysis method [17].

It must be pointed out that, from a view of the balance between local neuronal assemblies activities and global integrative processes upon which normal brain functions is primarily dependent [18], these researches mentioned above are all characterized with separately or isolatedly focusing on the effects of CHU either on regional brain activity or on a set of brain regions within a network. So, it is still difficult to generalize the findings of these separated studies for deep understanding heroin addiction mechanism.

At present, there are only a few studies dedicated to explore the heroin addiction mechanism from the view of altered balance or interrelationship between local neuronal reactivity and high-order neuronal network in heroin addicts’ brain. Daglish et al. [19] adopted H_2_^15^O position emission tomography (PET) to exam the drug-related cue induced change of regional cerebral blood flow and its correlation with craving score. And then by functional connectivity analysis they identified the neuronal circuitry involved in opiate craving. Liu et al. [20] analyzed the functional connectivity intensity of brain region in resting state networks of heroin dependence (HD) and correlated it with neural blood-oxygen-level dependent (BOLD) activation in the same brain region in task-related fMRI. These studies successfully identified several networks involving neural responses to drug-related cue from a task-resting view, a view of interaction between the specific brain regions having abnormal responses to external stimuli (operational) and its relevant networks under resting-state. Because there is still no theoretical interpretation made for the relationship between brain activity of resting and operational task so far [21], we think a rest-rest interaction view which addressing the relationship between the nature of segregated neuronal activity and their high-order neuronal organization pattern integrative in resting state can better reflect the nature of disease-involved neuronal activity.

Amplitude of low frequency fluctuation (ALFF), representing as the square root of the power spectrum in a low frequency range, was recently applied as a new biomarker for assessing brain physiopathological state by calculating the regional intensity of spontaneous fluctuation in BOLD signal in resting state [22]. Multiple psychological and neurological disorders were assessed with ALFF, such as attention deficits hyperactivity disorder [22], epilepsy [23] and Parkinson disease [24], demonstrating that ALFF is reliable approach to evaluate the nature and extent of physiopathological BOLD signal change of regional spontaneous neural activity (SNA) in resting-state [22], [25].

ALFF measurement was also used by Jiang et al. to investigate heroin-use-related SNA alteration in the HD individuals [26]. They found that compared with healthy controls, the ALFF of HD individuals was decreased in the dorsal anterior cingulated cortex (dACC), bilateral medial orbit frontal cortex, left dorsal lateral prefrontal cortex (dlPFC), left middle temporal gyrus and left cuneus, and was increased in the posterior and parietal regions, bilateral precuneus, bilateral angular, bilateral supramarginal gyrus and left posterior cingulated cortex (PCC). These findings were known brain regions contributing to psychological and mental presentation of heroin addicts which reported in previous studies [8], [27], indicating that ALFF measurement can characterize the nature and extent of SNA in HD brains. However, their study did not provide evidence for the correlation between CHU and SNA changes and not elucidate the altered balance between regional neuronal activity and brain integrative process in resting state in HD.

This study planed to investigate the relationship between heroin-use-induced disrupted local neural activity and its functional organization in resting state, by which we hope to explore the neural substrates contributing to the development of heroin addictive behavior pattern. We firstly used ALFF measurement to investigate the heroin-use-related altered baseline brain function of CHU patients, and then we identified which brain regions with changed SNA were correlated with CHU. After that, we applied region-based network analysis to provide a good visualization of heroin-use-induced functional connectivity properties of the brain regions with altered ALFF. We hypothesized that HD would have some brain regions with altered ALFF compared with normal controls (NC) and these regions would be mainly located in the prefrontal cortex and limbic system just as a previous study found. Meanwhile, we surmised that the altered ALFF value in these brain regions could be correlated with heroin use and these CHU-associated areas would be related to the dysfunctional connectivity of interacting brain subsystems in resting state.

## Materials and Methods

This present study was approved by Institutional Board of the Fourth Military Medical University, China, and conducted in accordance with Declaration of Helsinki.

### 2.1 Subjects

Seventeen HD patients (male, right-handed, mean age 33.9±6.3 years, age range 25∼42 years) were recruited from the Outpatient of Xi’an Methadone Substitution Treatment Center. From the very beginning they were on their own initiative to come to that Outpatient for seeking medical help and they all completed this study before receiving formal methadone maintenance treatment. By means of advertising we recruited fifteen male NC from local community, their mean age 34.3±7.3 years and age range 20∼43. They had not the history of drug use and were matched with the HD patients in the aspects of age, gender, right-handed, educational level and cigarette smoking (*p*>0.05). More detailed demographic information was given in Table 1.

**Table 1:** Demographic information of HD and NC.

Characteristics	NC (n = 15)	HD (n = 17)	*P* value
**Age (years)**	*34.3±7.3*	*33.9±6.3*	*p = 0.875*
**Education (years)**	*10.6±2.4*	*10.2±2.8*	*p = 0.699*
**Nicotine (no. cigarette/day)**	*18.3±2.1*	*20.0±6.1*	*p = 0.308*
**Duration of heroin use (months)**	*N/A*	*81.5±33.9*	
**Dosage of heroin use (g/day)**	*N/A*	*0.71±0.35*	

The inclusion criteria for the HD were as follows: a) they were screened by the Structural Clinical Interview (SCID-IV) at first and diagnosed as opioid dependence according to the diagnostic criteria of the DSM-IV-TR (American Psychiatric Association, 2002); b) their heroin use history was longer than 12 months and their mean daily dosage of heroin more than 0.3 g; 3) their urine test of heroin usage before methadone treatment was positive.

All the participants, NC or HD, would be excluded if they had any history of active neurological disease, a history of head trauma, medical disorders requiring immediate treatment, psychiatric disorders other than heroin dependence, and contraindications to MRI scan, or if they were taking any prescription drugs which might affect central nerves system within 1 week before MRI scanning. In accordance with the requirements of the Institutional Review Board of the Fourth Military Medical University, all the participants were fully informed of the details of the experiment and provided with the written consents for their involvement. All subjects finished this procedure before scanning.

### 2.2 Brain Imaging Methodology and Data Analysis

#### 2.2.1 MRI scanning

MRI data were acquired on a 3.0 T GE-Signa MRI scanner using an eight-channel head coil (GE Healthcare, Milwaukee, U.S.A.) in the Department of Radiology, Tangdu Hospital, the Fourth Military Medical University. Lying supine with head fixed by a belt and foam pads, subjects were instructed to keep their heads still, close their eyes and not to think anything specific. A routine structure MR scan was conducted to exclude gross cerebral pathology. After that, the resting-state fMRI data were abstained using T2*-weighted gradient-echo planar imaging (GRE-EPI) and single-shot gradient echo-echo planar imaging (EPI) pulse sequence, settings: 30 axial slices, TR = 2000 ms, TE = 30 ms, flip angle = 90°, FOV = 256 mm×256 mm, slice thickness = 5.0 mm, skip = 0 mm, Matrix = 64×64. This session lasted for 5 minutes and 10 seconds. And then, a high-resolution structure session was performed with contiguous slices to cover the whole brain in a steady state using an axial fast spoiled gradient recalled echo (3D-FSGPR) for anatomical reference, scanning parameters: TR = 7.8 ms, TE = 3.0 ms, TI = 450 ms, FOV = 256 mm×192 mm, slice thickness = 1.0 mm, skip = 0 mm, matrix = 256×256. After scanning, all subjects reported they were awake during all scans.

#### 2.2.2 Data preprocessing, ALFF calculation

The imaging data were mainly processed with SPM 8 (http://www.fil.ion.ucl.ac.uk/spm). The resting-state data were preprocessed by cutting the first five time points to eliminate nonequilibrium effects of magnetization and effect of fMRI scanning noise. The remaining time points (150 volumes) were used for fMRI analysis. For each subject, all functional images were realigned to the first volume. Translation and rotation were checked and subjects with head movements greater than 1 mm in any direction or head rotations greater than 1° were discarded. All the 17 HD and 15 NC participants met the movement criterion. Followed by spatially smoothed with a Gaussian Kernel (full width at half maximum = 8 mm) and spatial normalization to the Montreal Neurological Institute (MNI) template (Resampling voxel size = 3×3×3 mm^3^). The T1-weighted high-resolution image volume was also spatially normalized to the MNI template. All images spatially normalized to MNI template were transformed to Talairach and Tournoux coordinates and then filtered by using a band-passed filter (0.01 Hz–0.08 Hz) to reduce the effect of low-frequency drifts and high-frequency noise.

Further ALFF calculation was performed with REST software (http://resting-fmri.sourceforge.net) with an approach similar to that used in the previous study [26], [28]. In brief, after bandpass filtering (0.01–0.08 Hz) and linear-trend removal, the time series were transformed to the frequency domain using fast Fourier transform and the power spectrum was obtained. Since the power of a given frequency is proportional to the square of its amplitude in the original time series, the square root was calculated at each frequency of the power spectrum and then averaged across 0.01 to 0.08 Hz to yield a measure of ALFF from each voxel. For standardization purposes, this ALFF was then divided by the global mean ALFF value to standardize data across subjects analogous to approaches used in PET study [29].

Two-sample *t* test was used to assess the differences in age, education level, cigarette smoking between HD and NC group, *P*<0.05 being considered significant. The voxel-by-voxel-based ALFF comparisons between the two groups were also performed with two-sample *t* test (*P*<0.01, AlphaSim correction, cluster size>16). In addition, partial correlation was introduced to identify the association between ALFF alterations and heroin use after controlling for age, education, and nicotine usage in HD group (*P*<0.05). Here, the average values of ALFF in those brain regions of HD individuals where the ALFF values were significantly decreased or increased compared to the NC were considered as the ALFF alterations.

#### 2.2.3 Functional connectivity analysis

The resting-state properties in HD and NC were assessed using a seed-voxel-correlation-based functional connectivity method. We determined the “seeding” region as the brain regions where the ALFF value changed significantly between HC and NC group (diameter = 6 mm), identified the voxels with the largest difference from the NC’s in those regions, and then set the voxels as the centers to draw a sphere with a diameter of 6 mm, by which we obtained the regions of interest (ROIs) or the seeds. In current study, all ROIs with altered ALFF were used as “seeds” to explore the relationship between heroin-use-induced disrupted local neural activity and its functional organization in resting state as much as possible. The correlation between the seeds and the rest of the whole brain was analyzed in a voxel-wise manner by regressing out the effects of head motion parameters. The correlation coefficients were converted to an approximately normal distribution using Fisher’s z-transformation and then analyzed with a random effect one-sample *t*-test to identify voxels that showed a significantly positive correlation to the seed time series within HD and NC. The between-group comparison of z value maps between HD and NC was conducted with two-sample *t*-tests. Statistical significance was considered at *p*<0.05 (family-wise-error corrected) and *p*<0.01 (AlphaSim corrected, cluster>16).

## Results

### 3.1 ALFF Results and the Correlation between ALFF Alteration and Heroin Use

**Figure 1:**
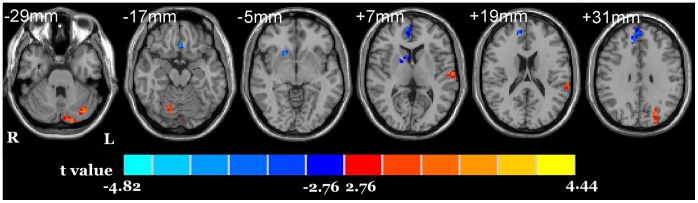
ALFF differences between HD and NC groups. The differences map from the bottom to the top, (every 12 mm), (p<0.01, corrected, T>2.7633 or <−2.7633, cluster size >16). Blue indicates HD individuals had decreased ALFF compared with NC and the red indicates the opposite.

**Figure 2:**
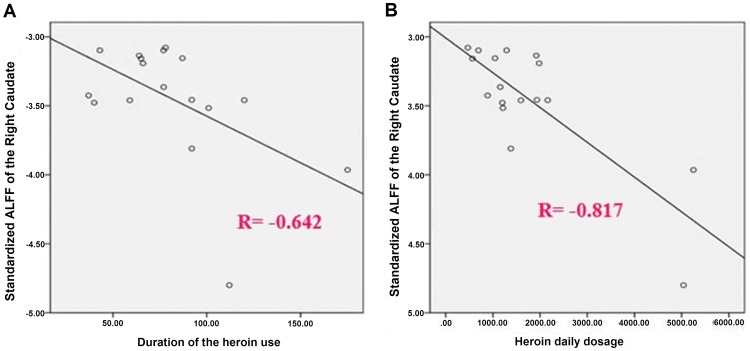
Bivariate scatter plots. A shows the negative correlation between the duration of the heroin use and the standardized ALFF of the right caudate, and B the negative correlation between the heroin daily dosage and the standardized ALFF of the right caudate.

Relative to NC, the HD group showed a significant decreased ALFF in the right caudate, right dACC (BA 32), right superior medial frontal cortex (BA 9, dlPFC) and a significant increased ALFF in the bilateral cerebellum, left superior temporal gyrus (BA 22) and left superior occipital gyrus (BA19) (Fig. 1). The ALFF value in the right caudate in HD individuals was negatively correlated with the duration of heroin use (r = −0.642, *p* = 0.010) and with the heroin daily dosage (r = −0.817, *p* = 0.000) (Fig. 2).

### 3.2 Functional Connectivity Results

Compared with NC, the HD showed reduced functional connectivity between the right caudate and the right superior temporal gyrus, bilateral middle frontal gyrus (BA 46, dlPFC), right superior frontal gyrus (BA 6), and left angular gyrus. Instead, an enhanced functional connectivity was found between the right caudate and the right cerebellum. The HD also showed increased functional connectivity of the right dACC with right lingual gyrus (BA 18), bilateral inferior frontal gyrus (BA 45,9), right inferior parietal lobule (BA 40). In addition, the functional connectivity of the right dACC in HD with several other brain regions was observed, including the bilateral middle temporal gyrus (BA21,38), left temporal gyrus (BA 21), right medial frontal gyrus (BA 10), left posterior frontal gyrus (BA 31) and left superior frontal gyrus (BA 8/6)(*p*<0.01, AlphaSim corrected, cluster>16). More details related sees Table 2.

**Table 2:** Differences in the functional connectivity of the regions with altered ALFF value between HD and NC (p<0.01, AlphaSim corrected, T>2.7633 or <−2.7633, cluster>16).

Seed		Brain Regions	BA	X	Y	Z	T-value	Cluster
**R Caudate (9, 12, 3)**	*R*	*Cerebellum*		*39*	−*69*	−*51*	*4.0046*	*39*
	*R*	*Superior Temporal Gyrus*		*57*	−*45*	*12*	−*4.5091*	*44*
	*R*	*Middle Frontal Gyrus*	*46*	*51*	*36*	*21*	−*4.2914*	*43*
	*L*	*Middle Frontal Gyrus*	*46*	−*51*	*30*	*24*	−*4.7831*	*38*
	*L*	*Angular Gyrus*	*40*	−*39*	−*48*	*33*	−*3.5858*	*32*
	*R*	*Superior Frontal Gyrus*	*6*	*27*	−*3*	*60*	−*3.4915*	*28*
**R Dorsal Anterior Cingulate Cortex** **(3, 48, 9)**	*R*	*Lingual Gyrus*	*18*	*18*	−*87*	−*9*	*5.965*	*48*
	*R*	*Inferior Frontal Gyrus*	*45*	*57*	*21*	*3*	*4.4644*	*23*
	*L*	*Inferior Frontal Gyrus*	*9*	−*39*	*33*	*45*	*4.3174*	*25*
	*R*	*Inferior Parietal Lobule*	*40*	*54*	−*42*	*45*	*4.3981*	*29*
	*L*	*Precentral Gyrus*	*6*	−*42*	*3*	*45*	*4.6489*	*34*
	*L*	*Middle Temporal Gyrus*	*21*	−*42*	*9*	−*42*	−*4.5096*	*28*
	*R*	*Middle Temporal Gyrus*	*38*	*36*	*12*	−*42*	−*4.685*	*18*
	*L*	*Inferior Temporal Gyrus*	*21*	−*63*	−*6*	−*18*	−*4.1035*	*21*
	*R*	*Medial Frontal Gyrus*	*10*	*9*	*57*	*3*	−*6.3597*	*107*
	*L*	*Posterior Frontal Gyrus*	*31*	−*12*	*48*	*12*	−*4.3827*	*60*
	*L*	*Superior Frontal Gyrus*	*8/6*	−*6*	*36*	*63*	−*4.3518*	*28*
**R Superior Medial Frontal Gyrus** **(6, 57, 30)**	*L*	*Precentral Gyrus*	*6*	−*42*	*0*	*42*	*3.8874*	*26*
	*R*	*Superior Frontal Gyrus*	*9*	*9*	*57*	*30*	−*5.2232*	*42*
**L Cerebellum** **(**−**9,** −**84,** −**27)**		*Anterior Cingulate*	*32*	*0*	*36*	−*6*	*3.7873*	*21*
	*R*	*Cuneus*	*18*	*18*	−*96*	*15*	−*3.4007*	*16*
	*L*	*Inferior Temporal Gyrus*	*37*	−*48*	−*48*	−*12*	−*3.4553*	*20*
	*L*	*Middle Occipital Gyrus*	*18*	−*12*	−*96*	*12*	−*3.4614*	*22*
	*R*	*Inferior Frontal Gyrus*		*48*	*30*	−*12*	−*3.687*	*17*
	*L*	*Superior Frontal Gyrus*	*9/46*	*46*	*35*	*33*	−*4.7454*	*45*
**L Superior Temporal Gyrus (**−**60,** −**30, 22)**	*L*	*Inferior Frontal Gyrus*	*47*	−*42*	*15*	−*6*	*3.3711*	*21*
	*R*	*Middle Frontal Gyrus*	*6*	*51*	*9*	*48*	*3.4194*	*28*
	*R*	*Superior Frontal Gyrus*		*6*	*5*	*37*	*3.9542*	*81*
	*L*	*Parahippocampa Gyrus*	*36*	−*30*	−*9*	−*33*	−*4.5746*	*41*
	*R*	*Parahippocampa Gyrus*	*36*	*30*	−*15*	*24*	−*4.5748*	*41*
	*L*	*Posterior Cingulate cortex*		−*18*	−*54*	*6*	−*3.3971*	*18*
	*L*	*Superior Frontal Gyrus*	*8*	−*25*	*33*	*54*	−*4.1690*	*65*
**L Superior Occiptal Gyrus** **(**−**24,** −**87, 33)**	*L*	*Caudate*		−*12*	*18*	*9*	*3.5670*	*19*
	*R*	*Inferior Occipital Gyrus*	*18/19*	*42*	−*81*	−*12*	−*3.7264*	*22*
	*R*	*Posterior Temporal Lobe*		*45*	−*75*	*27*	−*3.4564*	*19*

**Notes:** X, Y, Z: coordinates in the Talairach-Tournoux atlas. BA: Broadmann area. L, R: Left and right. T values from the *t*-test of a peak voxel (showing the greatest statistical differences within a cluster), a positive T value indicates increased functional connectivity in heroin dependent individuals.

## Discussion

This is the first study to explore the relationship between the CHU-induced disrupted local neural activities and its high-level functional organization pattern using a combined method of ALFF measurement and functional connectivity analysis. Consistent with previous study [26], this study also presents an extensive and systemic ALFF abnormalities in the cortical and subcortical regions of HD. Meanwhile, this study revealed via correlation analysis that only the ALFF value of right caudate had a negative correlation with heroin use duration and heroin daily dosage. Setting the brain regions with altered ALFF as the “seeding” region, we found the functional connectivity of right caudate with bilateral dlPFC, right superior frontal gyrus, left angular gyrus and right superior temporal was lower and the functional connectivity of right caudate with right cerebellum higher in HD. Furthermore, an opposite trend of functional connectivities between the right dACC, the posterior part of the left dlPFC and the left lateral PFC was observed. These findings may confirm the existence of hypoconnection of fronto-striatal circuitry, dysfunction of fronto-cerebellar circuitry, and abnormal lateral PFC-dACC connection in HD. These results support our hypothesis that the brain regions with ALFF alterations induced by CHU are associated with dysfunctional connectivity of interacting brain subsystems which may contribute to the process from first voluntary drug use to compulsive drug seeking and taking.

### 4.1 Decreased ALFF in Caudate

Caudate (dorsal striatum) is a principal brain region of mesostriatum Dopamine (DA) pathway and recognized to contribute to drug addiction [30]. Studies demonstrated that in the caudate existed significant DA changes when addicts were exposed to drug-related cues, and the magnitude of these changes were correlated with self-reports of craving [31]–[34]. All these disclose that the caudate is a crucial link between conditional and behavioral responses to procure drug and the DA activity responding to drug-related cues which mediates the habitual nature of subjective experience of caving and compulsive drug-seeking behaviors in addicts (habits development) [35]–[36]. Reportedly, impaired DA neuron and under-active DA system can be detected in the caudate of chronic opioid users [37]. Our correlation analysis revealed that only the ALFF value of right caudate has a negative correlation with heroin use, indicating that CHU may impair low-frequency oscillations of brain neuronal activity. In view of ALFF property, a convincing index to reflect the nature and extend of SNA, we suggest the changed ALFF in the caudate of HD might imply CHU-induced neural impairment and dopaminergic neuron function disruption.

In animal studies, the caudate was though to be involved in stimulus-response habit learning over the course of repeated cycles of drug-taking [38]. Volkow et al. observed the cue-elicited DA changes in caudate were associated with the estimates of addiction severity and the withdrawal symptoms scores in cocaine users, reflecting the strengthening of habits as chronicity of addiction progresses [31]–[32]. Thereby, we may consider that the reduced ALFF value in the caudate is a reflection of habitual behaviors of addiction, and its correlation with heroin use can reflect the strengthening of habit. Moreover, according to the postulation in ALFF abnormalities in heroin addicts [26], we think that the altered ALFF in caudate might be seen as a reliable neurobiological indicator of the habitual nature of drug-seeking behavior.

### 4.2 Increased ALFF Value in Cerebellum

Cerebellum is involved in addictive behavior [39]–[41]. Previous PET and fMRI studies observed that drug-conditioned cue elicited the increased metabolism and activation of cerebellum [40], [42]. When addicts performed reward expectation tasks, glucose metabolism was increased greatly in the cerebellum [43]. These suggest the cerebellum is also involved in drug-conditioned memories in addicts. And it has been noted and described that the cerebellum plays a compensatory role in inhibitory control [44] and decision-making behavior in addicts [45].

The cerebellum has many molecular targets where addictive substance can act on, such as glutamate receptor, dopamine receptor, cannabinoid receptor, etc [46]–[48]. The molecular and cellular actions of addictive drug on these receptors lead to the adaptive changes of neurotransmitter and intracellular signaling transduction pathway, and followed by reorganization and rewiring of cerebellum microzone [49]–[50]. It has been proposed that the drug-related neuroadaptation in cerebellum is associated with conditioned emotional memory, behavioral sensitization and perseverative behavioral phenotype transition, and all the associations contribute to the consolidation and performance of automatic goal-directed behavioral (habit) [39], [45], [51]–[52].

Our data demonstrated that, besides in the superior temporal gyrus and superior occipital gyrus in heroin users, the ALFF was also increased in the bilateral cerebellum. Based on this and the previous studies above, we argue that the altered ALFF in cerebellum might reflect the neuroadaptation and reorganization of cerebellar functional network caused by CHU, that there would be a correlation between ALFF alteration and addiction severity, and that the increase of ALFF value in cerebellum could be seen as an objective index for addiction degree assessment. These, of course, need further researches.

### 4.3 Functional Connectivity between Caudate and dlPFC

dlPFC is responsible for executive cognitive activities, such as focusing attention, self-control, working memory, planning in order to produce adaptive behavior automatically, and these activities are all robustly proven compromised previously in drug addiction through employing other methods [53]. In this present study, the functional connectivity between dlPFC and caudate was reduced in HD. We think this finding may implicate the dysfunction of fronto-striatal circuit in HD.

The dlPFC plays a role in the urge/craving of substance and behavioral addiction. For example, a previous fMRI study demonstrated that the activation of the dlPFC under drug cue corresponded to the cue-elicited craving in substance dependence [53]. And the resemble phenomenon was also found in pathological gamblers, online gaming addiction [54]–[55].

Reportedly, overnight abstinence from cigarette smoking was associated with higher brain activity in the dlPFC during a low demanding working memory load when compared with a non-deprivation state [56]. Cocaine addicts were observed having less cerebral blood flow in the dlPFC when performing decision-making task [57]. These suggest that when substance cues are presented and a positive expectancy is generated, the dlPFC may contribute to various processes in addiction, such as maintaining and coordinating the representations received from other regions during craving response, forming memory that is biased towards drug-related stimuli, inaccurate predictions or action planning, and choosing the immediate reward finally despite potential or future negative consequences.

Makris et al. found that a significant thinner dlPFC in addicts was associated with judgment and decision-making deficits when they compared the thickness of neocortical brain regions between cocaine-dependent and healthy control [58]. In the patients with internet addiction disorders, similar gray matter abnormalities in dlPFC were observed [59]. In addition, serial studies showed that the repeated high-frequency transcranial magnetic stimulation over the dlPFC reduced cue-induced craving and substance consumption, such as cigarette and food [60]–[61]. These findings reveal that dlPFC structure abnormalities are linked with the cognitive deficits and that impaired fronto-striatal connectivity is involved in the behavioral impairments and compulsive drug taking in addicts.

### 4.4 Functional Connectivity between Caudate and Cerebellum

Interestingly, our results demonstrated the functional connectivity between caudate and dlPFC was reduced but that between caudate and cerebellum was enhanced, implicating the functional reorganization of fronto-cerebellar circuitry. This happens to support an earlier proposal by Ito et al. that the prefrontal cortex and cerebellum work in parallel with a competitive manner [62]–[63]. Previous studies on drug addicts found that cerebellum activation correlated inversely with the prefrontal cortex activity when addicts were required to perform some cognitive tasks, such as GO-NOGO, IOWA gambling task or reward task [44]–[45], [64]. These indicate that the relevance of the cerebellum to external incentive stimuli might be enhanced when the prefrontal cortex are impaired by chronic drug use. It seems that the cerebellum control function in addicts is normally managed by the prefrontal cortex via repeated drug exposure. Ito et al. explained this procedure as internal models in cerebellum, namely a copy of the information is stored in the prefrontal and recruited when automatic processing and voluntary behavioral is required [62]–[63]. Evidently, the cerebellum is involved in drug-induced long-term emotional memory, behavioral sensitization and inflexible behavioral phenotype. Thereupon, we suggest that this reverse change of functional connectivity in fronto-cerebellar circuitry may reflect that cerebellar hyper-responsiveness is crucial for alleviating the prefrontal control on behavioral executive control and contributes to the transmission from instrumental behavior to habit. And we think that the functional connectivity between caudate and cerebellum might be taken as an objective index for addiction severity. Certainly it needs further verifications.

Our results also demonstrated that the functional connectivities of caudate with superior temporal gyrus, angular and superior frontal gyrus (BA 6) were reduced in HD, which may suggest a visual-spatial attention processing deficits in chronic heroin users. Researches have evidenced the existence of visual-spatial attention abnormalities in substance abusers which present with the ability to focus attention on drug-relevant stimuli and away from other alternatives [65]–[66].

### 4.5 Functional Connectivity of the Right dACC

It has been well documented that dACC and the posterior part of left dorsal lateral prefrontal cortex (dlPFC, BA6/8) are the key regions that contribute to top-down attention control and adjusting performance based on contextual demands [67]. Left dlPFC imposes a top-down attentional set and biasing which precedes late-stage selection performed by dACC [68]. dACC must enhance its activity to compensate to the low activity of left dlPFC when implementing top-down attentional control [69]. In the current study, the functional connectivity between the left superior frontal gyrus (BA 8/6) and dACC is decreased, indicating that heroin addicts may have difficulty in attentional allocation or attentional modulation. Meanwhile, the dACC is closely connected with the lateral PFC (BA9/46). When perceiving a conflict in a task, dACC impacts on allocation of attention and motor response in reward/cognition interactions by sending a signal to lateral PFC to achieve appropriate goal-directed behavior [27], [70]. Therefore, the altered functional connectivity between the left inferior frontal gyrus (BA 9), left superior frontal gyrus and dACC may suggest a disrupted attentional control and selection behavior, reflecting a neurophysiological substrate for characteristic pattern of behavior in heroin addicts.

The enhanced functional connectivities between the lingual gyrus, inferior parietal gyrus (BA 40) and right dACC are in line with the crucial role that dACC plays in voluntary attention and selective attention [71]. In heroin addicts, brain visual regions receive inputs from frontal and parietal areas, which biases sensory processing in favor of drug-related features [72]. The middle temporal gyrus (MT, BA21), dlPFC, PCC, temporal polar, dorsal medial frontal gyrus (dmMPFC) are the main components of human social brain [73]–[74]. So, a finding in this current study, the decreased functional connectivities of dACC with the right middle temporal gyrus (BA 38, temporal polar), the left middle temporal gyrus (BA 21), the left inferior temporal gyrus (BA 21), the right medial frontal Gyrus (BA 10, dmPFC), the left posterior frontal gyrus (BA 31, dPCC), may suggest the impaired ability of meaningful social interactions in heroin addicts.

Previous experiments demonstrated that the neuronal assemblies and their higher-level organization were a shot-term topological combination underlying multisensory integration, memory coding and retrieval during neuronal responses to external stimuli (drug cue) [19]–[20]. In contrast, our findings may reflect the CHU caused neuron mass self-assembling process which is closest to the real brain’s baseline activity. And from a view of the multivaribility and metastability of neuronal networks, these findings partly reveal a new relationship between CHU related neuronal assemblies and metastable pattern in resting state, which may explain the neural basis of compulsive behavioral phenotype in heroin addicts.

We’d like emphasize that our final results could be impacted by the limitations of only male subjects and no index used for measuring heroin addiction severity that could be correlated to the ALFF value or functional connectivity strength. We think it’s required to use an experiment designed with gender-matched group and clinical profiling to make a further verification in future. According to previous studies [26], [59], [75], we believe that the ALFF changes of dlPFC and dACC should be correlated with heroin use history. However, we did not find it from our ALFF analysis. We postulate this could be resulted from the relatively limited sample size of subjects. Therefore, for a more comprehensive understanding, a big sample size is also needed in the future experiment. It is also noteworthy that this current study detected the functional connectivities of the right superior medial frontal gyrus, left superior temporal gyrus, left superior occipital gyrus and left cerebellum in heroin addicts. Although these findings are hard to be interpreted as the reflection of drug-use-related changes of brain regions according to existing literature, they may be the new clues to explore the profound mystery of human brain and deserve more investigations in future.

### Conclusion

Using ALFF measurement and functional connectivity analysis, we demonstrate an altered balance between local neuronal assemblies activity and their integrated network organization patterns in chronic heroin users, verify that the brain regions with ALFF alterations induced by CHU are associated with dysfunctional connectivity of interacting brain subsystems. Moreover, our results may disclose the dysfunction of fronto-striatal circuitry and the dysfunction of fronto-cerebellar circuitry elicited by CHU in HD individuals, and the latter is in favor of Ito’s earlier theory about internal model in cerebellum and its contribution to the transition from instrumental behavioral to habit in addiction. These, we hope, might cast new light on the mechanisms underlying heroin addiction.

## References

[1] Everitt BJ, Robbins TW (2005) Neural systems of reinforcement for drug addiction: from actions to habits to compulsion. Nat Neurosci 8: 1481–1489. 16251991 10.1038/nn1579

[2] Yin HH, Knowlton BJ (2006) The role of the basal ganglia in habit formation. Nat Rev Neurosci 7: 464–476. 16715055 10.1038/nrn1919

[3] Volkow ND, Wang GJ, Fowler JS, Tomasi D, Telang F (2011) Addiction: beyond dopamine reward circuitry. Proc Natl Acad Sci U S A 108: 15037–15042. 21402948 10.1073/pnas.1010654108 PMC3174598

[4] Lu L, Fang Y, Wang X (2008) Drug abuse in China: past, present and future. Cell Mol Neurobiol 28: 479–490. 17990098 10.1007/s10571-007-9225-2 PMC11515005

[5] Liu H, Li L, Hao Y, Cao D, Xu L, et al (2008) Disrupted white matter integrity in heroin dependence: a controlled study utilizing diffusion tensor imaging. Am J Drug Alcohol Abuse 34: 562–575. 18720268 10.1080/00952990802295238

[6] Bora E, Yucel M, Fornito A, Pantelis C, Harrison BJ, et al.. (2010) White matter microstructure in opiate addiction. Addict Biol. 10.1111/j.1369-1600.2010.00266.x 21070508

[7] Wang X, Zhou X, Liao Y, Tang J, Liu T, et al (2011) Microstructural disruption of white matter in heroin addicts revealed by diffusion tensor imaging: a controlled study. Zhong Nan Da Xue Xue Bao Yi Xue Ban 36: 728–732. 21937797 10.3969/j.issn.1672-7347.2011.08.005

[8] Liu H, Hao Y, Kaneko Y, Ouyang X, Zhang Y, et al (2009) Frontal and cingulate gray matter volume reduction in heroin dependence: optimized voxel-based morphometry. Psychiatry Clin Neurosci 63: 563–568. 19531112 10.1111/j.1440-1819.2009.01989.x

[9] Yuan Y, Zhu Z, Shi J, Zou Z, Yuan F, et al (2009) Gray matter density negatively correlates with duration of heroin use in young lifetime heroin-dependent individuals. Brain Cogn 71: 223–228. 19775795 10.1016/j.bandc.2009.08.014

[10] Fu LP, Bi GH, Zou ZT, Wang Y, Ye EM, et al (2008) Impaired response inhibition function in abstinent heroin dependents: an fMRI study. Neurosci Lett 438: 322–326. 18485592 10.1016/j.neulet.2008.04.033

[11] Lee TM, Zhou WH, Luo XJ, Yuen KS, Ruan XZ, et al (2005) Neural activity associated with cognitive regulation in heroin users: A fMRI study. Neurosci Lett 382: 211–216. 15925092 10.1016/j.neulet.2005.03.053

[12] Xiao Z, Lee T, Zhang JX, Wu Q, Wu R, et al (2006) Thirsty heroin addicts show different fMRI activations when exposed to water-related and drug-related cues. Drug Alcohol Depend 83: 157–162. 16406379 10.1016/j.drugalcdep.2005.11.012

[13] Pruessner JC, Champagne F, Meaney MJ, Dagher A (2004) Dopamine release in response to a psychological stress in humans and its relationship to early life maternal care: a positron emission tomography study using [11C]raclopride. J Neurosci 24: 2825–2831. 15028776 10.1523/JNEUROSCI.3422-03.2004 PMC6729514

[14] Baler RD, Volkow ND (2006) Drug addiction: the neurobiology of disrupted self-control. Trends Mol Med 12: 559–566. 17070107 10.1016/j.molmed.2006.10.005

[15] Liu J, Liang J, Qin W, Tian J, Yuan K, et al (2009) Dysfunctional connectivity patterns in chronic heroin users: an fMRI study. Neurosci Lett 460: 72–77. 19450664 10.1016/j.neulet.2009.05.038

[16] Ma N, Liu Y, Li N, Wang CX, Zhang H, et al (2010) Addiction related alteration in resting-state brain connectivity. Neuroimage 49: 738–744. 19703568 10.1016/j.neuroimage.2009.08.037 PMC2764798

[17] Ma N, Liu Y, Fu XM, Li N, Wang CX, et al (2011) Abnormal brain default-mode network functional connectivity in drug addicts. PLoS One 6: e16560. 21298074 10.1371/journal.pone.0016560 PMC3027699

[18] Fingelkurts AA (2004) Making complexity simpler: multivariability and metastability in the brain. Int J Neurosci 114: 843–862. 15204050 10.1080/00207450490450046

[19] Daglish MR, Weinstein A, Malizia AL, Wilson S, Melichar JK, et al (2003) Functional connectivity analysis of the neural circuits of opiate craving: “more” rather than “different”? Neuroimage 20: 1964–1970. 14683702 10.1016/j.neuroimage.2003.07.025

[20] Liu J, Qin W, Yuan K, Li J, Wang W, et al (2011) Interaction between Dysfunctional Connectivity at Rest and Heroin Cues-Induced Brain Responses in Male Abstinent Heroin-Dependent Individuals. PLoS One 6: e23098. 22028765 10.1371/journal.pone.0023098 PMC3196491

[21] Morcom AM, Fletcher PC (2007) Does the brain have a baseline? Why we should be resisting a rest. Neuroimage 37: 1073–1082. 10.1016/j.neuroimage.2007.06.019 17681817

[22] Zang YF, He Y, Zhu CZ, Cao QJ, Sui MQ, et al (2007) Altered baseline brain activity in children with ADHD revealed by resting-state functional MRI. Brain Dev 29: 83–91. 16919409 10.1016/j.braindev.2006.07.002

[23] Zhang ZQ, Lu GM, Zhong Y, Tan QF, Zhu JG, et al (2008) [Application of amplitude of low-frequency fluctuation to the temporal lobe epilepsy with bilateral hippocampal sclerosis: an fMRI study]. Zhonghua Yi Xue Za Zhi 88: 1594–1598. 19035096

[24] Skidmore FM, Yang M, Baxter L, von Deneen K, Collingwood J, et al.. (2011) Apathy, depression, and motor symptoms have distinct and separable resting activity patterns in idiopathic Parkinson disease. Neuroimage. 10.1016/j.neuroimage.2011.07.012 21782030

[25] Yang H, Long XY, Yang Y, Yan H, Zhu CZ, et al (2007) Amplitude of low frequency fluctuation within visual areas revealed by resting-state functional MRI. Neuroimage 36: 144–152. 17434757 10.1016/j.neuroimage.2007.01.054

[26] Jiang GH, Qiu YW, Zhang XL, Han LJ, Lv XF, et al (2011) Amplitude low-frequency oscillation abnormalities in the heroin users: a resting state fMRI study. Neuroimage 57: 149–154. 21515385 10.1016/j.neuroimage.2011.04.004

[27] Bush G, Luu P, Posner MI (2000) Cognitive and emotional influences in anterior cingulate cortex. Trends Cogn Sci 4: 215–222. 10827444 10.1016/s1364-6613(00)01483-2

[28] Zhang Z, Lu G, Zhong Y, Tan Q, Chen H, et al (2010) fMRI study of mesial temporal lobe epilepsy using amplitude of low-frequency fluctuation analysis. Hum Brain Mapp 31: 1851–1861. 20225278 10.1002/hbm.20982 PMC6870704

[29] Raichle ME, MacLeod AM, Snyder AZ, Powers WJ, Gusnard DA, et al (2001) A default mode of brain function. Proc Natl Acad Sci U S A 98: 676–682. 11209064 10.1073/pnas.98.2.676 PMC14647

[30] Wise RA (2009) Roles for nigrostriatal–not just mesocorticolimbic–dopamine in reward and addiction. Trends Neurosci 32: 517–524. 19758714 10.1016/j.tins.2009.06.004 PMC2755633

[31] Volkow ND, Wang GJ, Telang F, Fowler JS, Logan J, et al (2006) Cocaine cues and dopamine in dorsal striatum: mechanism of craving in cocaine addiction. J Neurosci 26: 6583–6588. 16775146 10.1523/JNEUROSCI.1544-06.2006 PMC6674019

[32] Volkow ND, Wang GJ, Fowler JS, Tomasi D, Telang F (2011) Addiction: beyond dopamine reward circuitry. Proc Natl Acad Sci U S A 108: 15037–15042. 21402948 10.1073/pnas.1010654108 PMC3174598

[33] Sinha R, Lacadie C, Skudlarski P, Fulbright RK, Rounsaville BJ, et al (2005) Neural activity associated with stress-induced cocaine craving: a functional magnetic resonance imaging study. Psychopharmacology (Berl) 183: 171–180. 16163517 10.1007/s00213-005-0147-8

[34] Garavan H, Kelley D, Rosen A, Rao SM, Stein EA (2000) Practice-related functional activation changes in a working memory task. Microsc Res Tech 51: 54–63. 11002353 10.1002/1097-0029(20001001)51:1<54::AID-JEMT6>3.0.CO;2-J

[35] Vollstadt-Klein S, Wichert S, Rabinstein J, Buhler M, Klein O, et al (2010) Initial, habitual and compulsive alcohol use is characterized by a shift of cue processing from ventral to dorsal striatum. Addiction 105: 1741–1749. 20670348 10.1111/j.1360-0443.2010.03022.x

[36] Belin D, Everitt BJ (2008) Cocaine seeking habits depend upon dopamine-dependent serial connectivity linking the ventral with the dorsal striatum. Neuron 57: 432–441. 18255035 10.1016/j.neuron.2007.12.019

[37] Shi J, Zhao LY, Copersino ML, Fang YX, Chen Y, et al (2008) PET imaging of dopamine transporter and drug craving during methadone maintenance treatment and after prolonged abstinence in heroin users. Eur J Pharmacol 579: 160–166. 17977528 10.1016/j.ejphar.2007.09.042

[38] Vanderschuren LJ, Di Ciano P, Everitt BJ (2005) Involvement of the dorsal striatum in cue-controlled cocaine seeking. J Neurosci 25: 8665–8670. 16177034 10.1523/JNEUROSCI.0925-05.2005 PMC6725521

[39] Anderson CM, Maas LC, Frederick B, Bendor JT, Spencer TJ, et al (2006) Cerebellar vermis involvement in cocaine-related behaviors. Neuropsychopharmacology 31: 1318–1326. 16237382 10.1038/sj.npp.1300937

[40] Grant S, London ED, Newlin DB, Villemagne VL, Liu X, et al (1996) Activation of memory circuits during cue-elicited cocaine craving. Proc Natl Acad Sci U S A 93: 12040–12045. 8876259 10.1073/pnas.93.21.12040 PMC38179

[41] Schneider F, Habel U, Wagner M, Franke P, Salloum JB, et al (2001) Subcortical correlates of craving in recently abstinent alcoholic patients. Am J Psychiatry 158: 1075–1083. 11431229 10.1176/appi.ajp.158.7.1075

[42] Wang GJ, Volkow ND, Fowler JS, Cervany P, Hitzemann RJ, et al (1999) Regional brain metabolic activation during craving elicited by recall of previous drug experiences. Life Sci 64: 775–784. 10075110 10.1016/s0024-3205(98)00619-5

[43] Volkow ND, Wang GJ, Ma Y, Fowler JS, Zhu W, et al (2003) Expectation enhances the regional brain metabolic and the reinforcing effects of stimulants in cocaine abusers. J Neurosci 23: 11461–11468. 14673011 10.1523/JNEUROSCI.23-36-11461.2003 PMC6740524

[44] Bolla KI, Eldreth DA, Matochik JA, Cadet JL (2005) Neural substrates of faulty decision-making in abstinent marijuana users. Neuroimage 26: 480–492. 15907305 10.1016/j.neuroimage.2005.02.012

[45] Hester R, Garavan H (2004) Executive dysfunction in cocaine addiction: evidence for discordant frontal, cingulate, and cerebellar activity. J Neurosci 24: 11017–11022. 15590917 10.1523/JNEUROSCI.3321-04.2004 PMC6730277

[46] Gruol DL, Parsons KL, DiJulio N (1997) Acute ethanol alters calcium signals elicited by glutamate receptor agonists and K+ depolarization in cultured cerebellar Purkinje neurons. Brain Res 773: 82–89. 9409708 10.1016/s0006-8993(97)00912-8

[47] Botta P, Radcliffe RA, Carta M, Mameli M, Daly E, et al (2007) Modulation of GABAA receptors in cerebellar granule neurons by ethanol: a review of genetic and electrophysiological studies. Alcohol 41: 187–199. 17521847 10.1016/j.alcohol.2007.04.004 PMC1986723

[48] Thanos PK, Dimitrakakis ES, Rice O, Gifford A, Volkow ND (2005) Ethanol self-administration and ethanol conditioned place preference are reduced in mice lacking cannabinoid CB1 receptors. Behav Brain Res 164: 206–213. 16140402 10.1016/j.bbr.2005.06.021

[49] Beck CH (1995) Acute treatment with antidepressant drugs selectively increases the expression of c-fos in the rat brain. J Psychiatry Neurosci 20: 25–32. 7865498 PMC1188655

[50] Jimenez-Rivera CA, Segarra O, Jimenez Z, Waterhouse BD (2000) Effects of intravenous cocaine administration on cerebellar Purkinje cell activity. Eur J Pharmacol 407: 91–100. 11050295 10.1016/s0014-2999(00)00711-1

[51] Rubino T, Vigano D, Massi P, Parolaro D (2003) Cellular mechanisms of Delta 9-tetrahydrocannabinol behavioural sensitization. Eur J Neurosci 17: 325–330. 12542669 10.1046/j.1460-9568.2003.02452.x

[52] Miquel M, Toledo R, Garcia LI, Coria-Avila GA, Manzo J (2009) Why should we keep the cerebellum in mind when thinking about addiction? Curr Drug Abuse Rev 2: 26–40. 19630735 10.2174/1874473710902010026

[53] Garavan H, Pankiewicz J, Bloom A, Cho JK, Sperry L, et al (2000) Cue-induced cocaine craving: neuroanatomical specificity for drug users and drug stimuli. Am J Psychiatry 157: 1789–1798. 11058476 10.1176/appi.ajp.157.11.1789

[54] Crockford DN, Goodyear B, Edwards J, Quickfall J, el-Guebaly N (2005) Cue-induced brain activity in pathological gamblers. Biol Psychiatry 58: 787–795. 15993856 10.1016/j.biopsych.2005.04.037

[55] Ko CH, Liu GC, Hsiao S, Yen JY, Yang MJ, et al (2009) Brain activities associated with gaming urge of online gaming addiction. J Psychiatr Res 43: 739–747. 18996542 10.1016/j.jpsychires.2008.09.012

[56] Xu J, Mendrek A, Cohen MS, Monterosso J, Rodriguez P, et al (2005) Brain activity in cigarette smokers performing a working memory task: effect of smoking abstinence. Biol Psychiatry 58: 143–150. 16038685 10.1016/j.biopsych.2005.03.028 PMC2773671

[57] Bolla KI, Eldreth DA, London ED, Kiehl KA, Mouratidis M, et al (2003) Orbitofrontal cortex dysfunction in abstinent cocaine abusers performing a decision-making task. Neuroimage 19: 1085–1094. 12880834 10.1016/s1053-8119(03)00113-7 PMC2767245

[58] Makris N, Gasic GP, Kennedy DN, Hodge SM, Kaiser JR, et al (2008) Cortical thickness abnormalities in cocaine addiction–a reflection of both drug use and a pre-existing disposition to drug abuse? Neuron 60: 174–188. 18940597 10.1016/j.neuron.2008.08.011 PMC3772717

[59] Yuan K, Qin W, Wang G, Zeng F, Zhao L, et al (2011) Microstructure abnormalities in adolescents with internet addiction disorder. PLoS One 6: e20708. 21677775 10.1371/journal.pone.0020708 PMC3108989

[60] Amiaz R, Levy D, Vainiger D, Grunhaus L, Zangen A (2009) Repeated high-frequency transcranial magnetic stimulation over the dorsolateral prefrontal cortex reduces cigarette craving and consumption. Addiction 104: 653–660. 19183128 10.1111/j.1360-0443.2008.02448.x

[61] Van den Eynde F, Claudino AM, Mogg A, Horrell L, Stahl D, et al (2010) Repetitive transcranial magnetic stimulation reduces cue-induced food craving in bulimic disorders. Biol Psychiatry 67: 793–795. 20060105 10.1016/j.biopsych.2009.11.023

[62] Ito M (2008) Control of mental activities by internal models in the cerebellum. Nat Rev Neurosci 9: 304–313. 18319727 10.1038/nrn2332

[63] Ito M (2006) Cerebellar circuitry as a neuronal machine. Prog Neurobiol 78: 272–303. 16759785 10.1016/j.pneurobio.2006.02.006

[64] Martin-Solch C, Magyar S, Kunig G, Missimer J, Schultz W, et al (2001) Changes in brain activation associated with reward processing in smokers and nonsmokers. A positron emission tomography study. Exp Brain Res 139: 278–286. 11545466 10.1007/s002210100751

[65] Oscar-Berman M, Ellis RJ (1987) Cognitive deficits related to memory impairments in alcoholism. Recent Dev Alcohol 5: 59–80. 3550918 10.1007/978-1-4899-1684-6_3

[66] Ahveninen J, Jaaskelainen IP, Pekkonen E, Hallberg A, Hietanen M, et al (2000) Increased distractibility by task-irrelevant sound changes in abstinent alcoholics. Alcohol Clin Exp Res 24: 1850–1854. 11141044

[67] Banich MT, Milham MP, Atchley RA, Cohen NJ, Webb A, et al (2000) Prefrontal regions play a predominant role in imposing an attentional ‘set’: evidence from fMRI. Brain Res Cogn Brain Res 10: 1–9. 10978687 10.1016/s0926-6410(00)00015-x

[68] Banich MT, Burgess GC, Depue BE, Ruzic L, Bidwell LC, et al (2009) The neural basis of sustained and transient attentional control in young adults with ADHD. Neuropsychologia 47: 3095–3104. 19619566 10.1016/j.neuropsychologia.2009.07.005 PMC3703501

[69] Silton RL, Heller W, Towers DN, Engels AS, Spielberg JM, et al (2010) The time course of activity in dorsolateral prefrontal cortex and anterior cingulate cortex during top-down attentional control. Neuroimage 50: 1292–1302. 20035885 10.1016/j.neuroimage.2009.12.061 PMC5998337

[70] Davidson RJ, Pizzagalli D, Nitschke JB, Putnam K (2002) Depression: perspectives from affective neuroscience. Annu Rev Psychol 53: 545–574. 11752496 10.1146/annurev.psych.53.100901.135148

[71] Weissman DH, Mangun GR, Woldorff MG (2002) A role for top-down attentional orienting during interference between global and local aspects of hierarchical stimuli. Neuroimage 17: 1266–1276. 12414266 10.1006/nimg.2002.1284

[72] Cox WM, Fadardi JS, Pothos EM (2006) The addiction-stroop test: Theoretical considerations and procedural recommendations. Psychol Bull 132: 443–476. 16719569 10.1037/0033-2909.132.3.443

[73] Brothers L, Ring B, Kling A (1990) Response of neurons in the macaque amygdala to complex social stimuli. Behav Brain Res 41: 199–213. 2288672 10.1016/0166-4328(90)90108-q

[74] Tavares P, Lawrence AD, Barnard PJ (2008) Paying attention to social meaning: an FMRI study. Cereb Cortex 18: 1876–1885. 18065722 10.1093/cercor/bhm212

[75] Bolla K, Ernst M, Kiehl K, Mouratidis M, Eldreth D, et al (2004) Prefrontal cortical dysfunction in abstinent cocaine abusers. J Neuropsychiatry Clin Neurosci 16: 456–464. 15616172 10.1176/appi.neuropsych.16.4.456 PMC2771441

